# Supplementary material for: Construction of Chemically Bonded Interface of Organic/Inorganic g-C3N4/LDH Heterojunction for Z-Schematic Photocatalytic H2 Generation
Source: Nanomaterials (Basel). 2021 Oct 18;11(10):2762. doi: 10.3390/nano11102762 (PMC8539041; doi:10.3390/nano11102762)
Supplement: Supplementary file 1 [file nanomaterials-11-02762-s001.zip › nanomaterials-1415208-supplementary.pdf]

Supporting Information

Construction of Chemically Bonded Interface of Organic/Inorganic g-C_3N_4/LDH Heterojunction for Z-Schematic Photocatalytic H_2 Generation

Yuzhou Xia [1], Ruowen Liang [1], Min-Quan Yang [2,*], Shuying Zhu [3,*] and Guiyang Yan [1, 4,*]

[1] Fujian Province University Key Laboratory of Green Energy and Environment Catalysis, Ningde Normal University, Ningde, Fujian, 352100, P. R. China
[2] College of Environmental Science and Engineering, Fujian Key Laboratory of Pollution Control & Resource Reuse, Fujian Normal University, Fuzhou 350007, P. R. China
[3] College of Chemistry, Fuzhou University, Fuzhou 350116, P. R. China
[4] Provincial Key Laboratory of Featured Materials in Biochemical Industry, Ningde Normal University, Ningde, Fujian, 352100, P. R. China
* Correspondence: yangmq@fjnu.edu.cn (Prof. Min-Quan Yang), syzhu@fzu.edu.cn (Dr. Shuying Zhu) and ygyfjnu@163.com (Prof. Guiyang Yan)

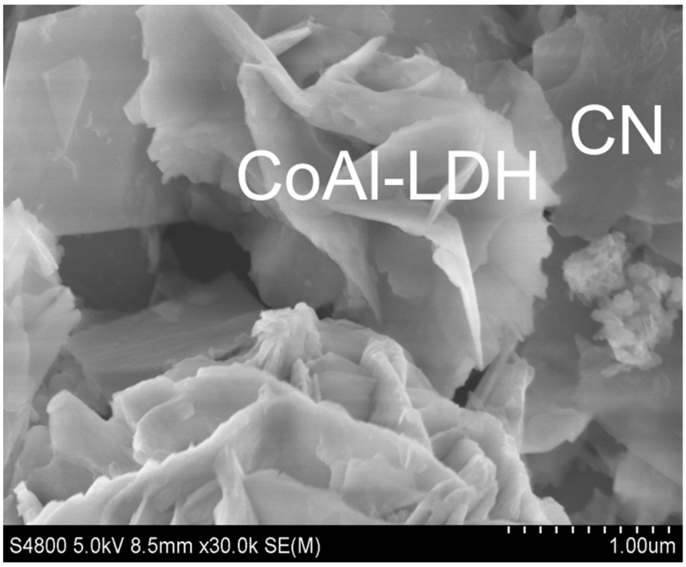

Figure S1. SEM image of the prepared CN-$CoAl_{0.7}$.

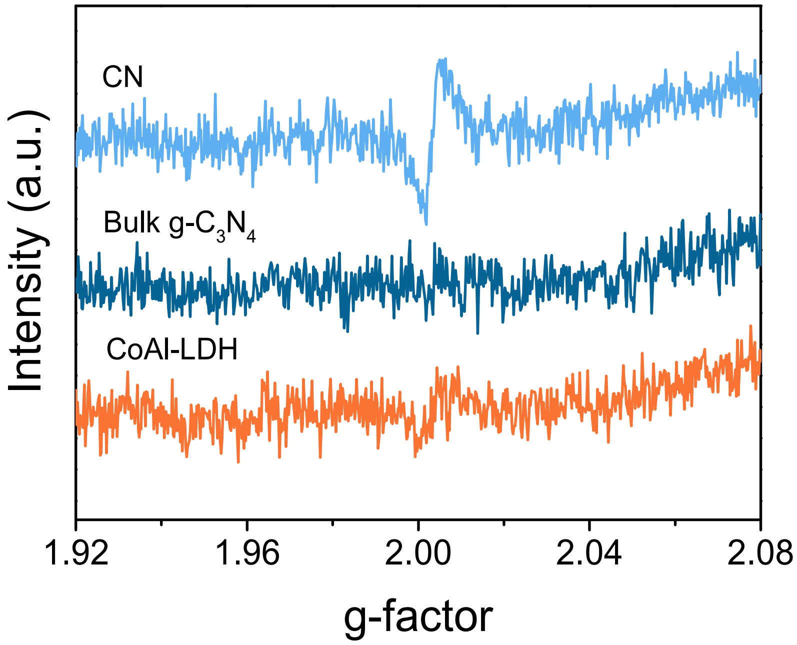

Figure S2. EPR spectra of bulk g-C_3N_4, CN and CoAl-LDH.

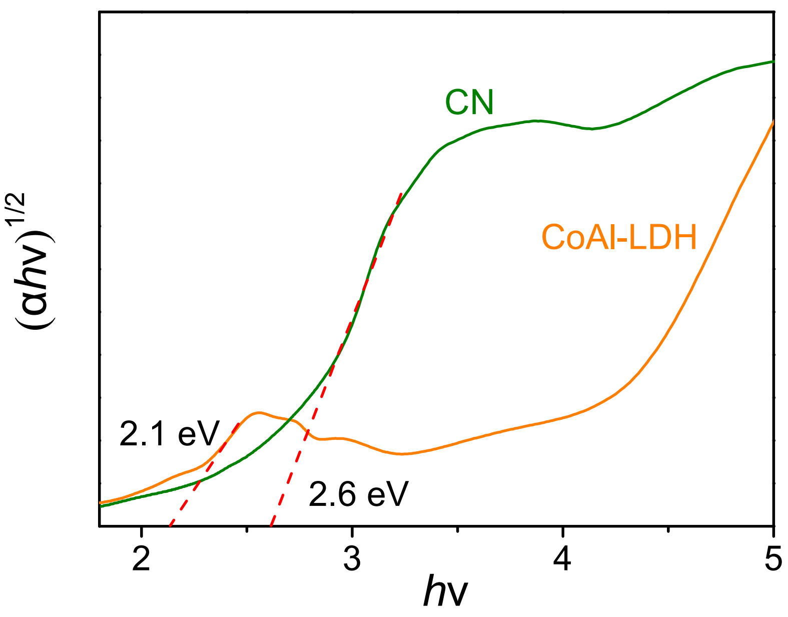

Figure S3. Tauc plots of CN and CoAl-LDH.

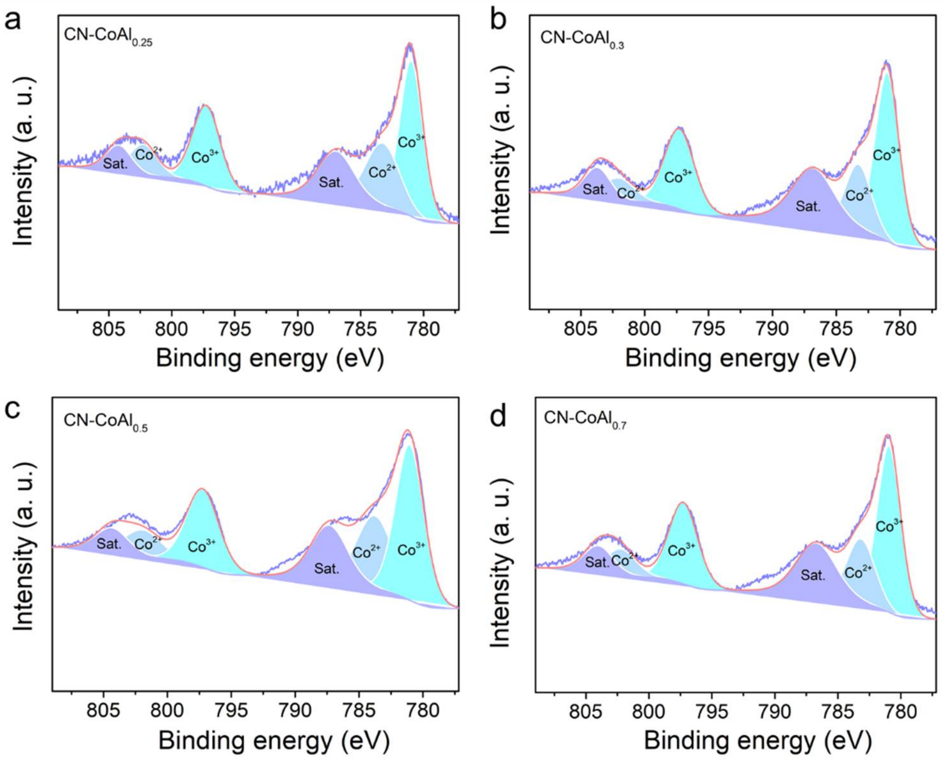

Figure S4. XPS spectra of Co in CN-CoAlx samples CN-CoAl$_{0.25}$ (a), CN-CoAl$_{0.3}$ (b), CN-CoAl$_{0.5}$ (c) and CN-CoAl$_{0.7}$ (d).

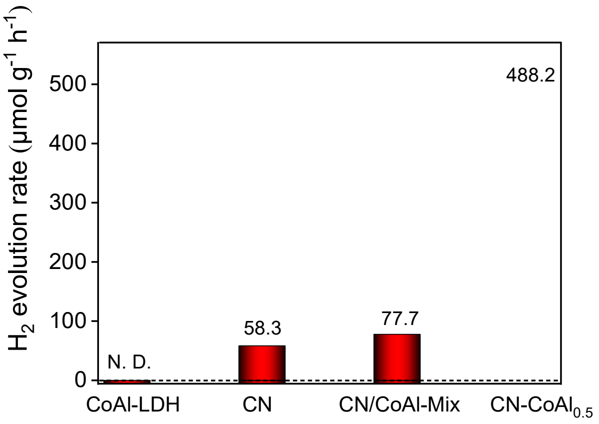

Figure S5. Photocatalytic H_2 evolution rates of CoAl-LDH, CN, CN/CoAl-Mix and CN-CoAl$_{0.5}$.

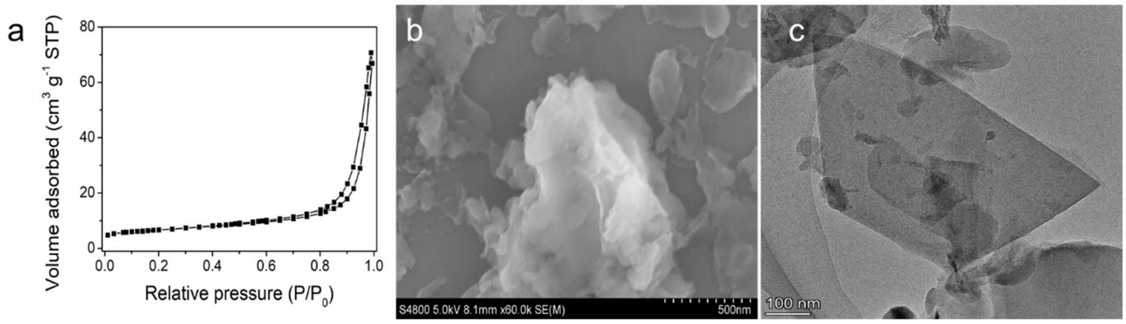

Figure S6. BET (a), SEM (b) and TEM (c) analyses of CN-CoAl$_{0.5}$ after 5 cycles of H_2-generation stability text.
